# Genomic Insight into *Shimazuella Soli* Sp. Nov. Isolated from Soil and Its Putative Novel Class II Lasso Peptide

**DOI:** 10.3390/bioengineering9120812

**Published:** 2022-12-16

**Authors:** Chun-Zhi Jin, Jong Min Lee, Chang-Jin Kim, Hyung-Gwan Lee, Kee-Sun Shin

**Affiliations:** 1 Cell Factory Research Center, Korea Research Institute of Bioscience and Biotechnology (KRIBB), 125 Gwahak-ro, Yuseong-gu, Daejeon 34141, Republic of Korea; 2 Department of Biotechnology, Pukyong National University, 45 Yongso-ro, Nam-gu, Busan 48513, Republic of Korea; 3 Industrial Biomaterial Research Center, Korea Research Institute of Bioscience and Biotechnology (KRIBB), 125 Gwahak-ro, Yuseong-gu, Daejeon 34141, Republic of Korea

**Keywords:** *Shimazuella soli* sp. nov., polyphasic taxonomy, lasso peptide, genome mining

## Abstract

The strain designated as AN120528^T^ was isolated from farmland soil in South Korea. This strain grows well on R2A medium at 28 °C. The cells are an off-white colour and have no hyphae. The phylogenetic analysis indicated that the strain is a member of the genus *Shimazuella* with a 98.11% similarity to *Shimazuella alba* KC615^T^ and a 97.05% similarity to *S*. *kribbensis* KCTC 9933^T^, respectively. The strain AN120528^T^ shares common chemotaxonomic features with the other two type strains in the genus. It has MK-9 (H_4_) and MK-10 (H_4_) as its predominant menaquinones. The major fatty acids are *iso*-C_14:0_, *iso*-C_15:0_, *anteiso*-C_15:0_ and *iso*-C_16:0_. *Diphosphatidylglycerol* (DPG), *phosphatidylethanolamine* (PE), *phosphatidylglycerol* (PG), *lipids* (L), and *aminolipids* (AL) were identified as the major cellular polar lipids. Analysis of the peptidoglycan showed the presence of meso-diaminopimelic acid. Whole-genome sequencing revealed that the genome of the strain is approximately 3.3 Mbp in size. The strain showed a 77.5% average nucleotide identity (ANI) with *S*. *alba* KC615^T^. The genomic DNA (gDNA) G + C content is 39.0%. Based on polyphasic taxonomy analysis, it is proposed that this strain, AN120528^T^, represents a novel species in the genus *Shimazuella*, designated as *Shimazuella soli* sp. nov. The type stain is AN120528^T^ (=KCTC 39810^T^ = DSM 103571^T^). Furthermore, shimazuellin I, a new 15-amino-acid peptide, was discovered in the AN120528^T^ through genome mining; it has the features of a lasso peptide, containing eight amino acids (-G-Q-G-G-S-N-N-D-) that form a macrolactam ring and seven amino acids (-D-G-W-Y-H-S-K-) that form a tail.

## 1. Introduction

The genus *Shimazuella* belongs to the family *Thermoactinomycetaceae*, order *Caryophanales*, class *Bacilli* and phylum *Bacillota* [1,2]. The genus only had two valid published species [3], with *Shimazuella kribbensis* as the type species and *Shimazuella alba*, which was proposed as a new member. *Shimazuella* spp. are all gram-positive, aerobic, mesophilic and have white-coloured cells [2,4]. The two species, *S*. *kribbensis* and *S*. *alba*, are both isolated from soil. The cells can grow on International Streptomyces Project medium number 2 (ISP 2), 3 (ISP 3) and nutrient media. Based on chemotaxonomic study, *Shimazuella* contains MK-9 (H_4_) and MK-10 (H_4_) as predominant menaquinones and PE as a major polar lipid. The gDNA G+C contents are between 38.5 and 39.4%, and the genome size is about 3.98 Mbp. The major fatty acids are *anteiso*-C_15:0_ for both, *iso*-C_16:0_, C_16:0_, *iso*-C_15:0_ and *anteiso*-C_17:0_ for *S. kribbensis* and C_20:0_ and C_18:0_ for *S. alba* [2,4]. Both species have ribose and glucose in their cell-wall hydrolysates. In addition, *Shimazuella* spp. can grow on media with a pH of 6.0–8.0 at temperature range of 28–37 °C and can tolerate NaCl up to 1%.

Bioactive microbial peptides are short fragments of proteins produced by microorganisms and have been revealed to have substantial potential as drugs that maintain physiological homeostasis, such as antimicrobials, antioxidants, gut homeostasis therapies and immunomodulators [5]. Lasso peptides in bacteria are ribosomally synthesised and post-translationally modified peptides (RiPPs) with a unique N-terminal macrolactam ring structure and a C-terminal linear tail [6]. The peptides can be classified into four types according to the number of disulfide bonds. Among them, class II lasso peptides feature no disulfide bridges, and the topology is stabilised by steric interactions. Class II lasso peptides are known for their notable biological activities, including their antimicrobial, peptide antagonist, protease inhibitory and anti-cancer activities [7]. This wide range of application potential motivates researchers to find novel lasso peptides using genome mining. However, the detailed mechanism of maturation of the peptide has remained elusive due to the lack of structural information about the enzymes involved [6].

During our investigation on the microbial diversity of soil in Korea, we isolated a novel species belonging to the genus *Shimazuella*. In this study, we describe the characteristics of a novel species in the genus *Shimazuella* identified through polyphasic taxonomy analysis. We also report a novel class II lasso peptide, biosynthetic gene clusters (BGCs) and the inferred biosynthesis mechanism revealed using genome mining.

## 2. Materials and Methods

### 2.1. The Media and Reagents

All media used for bacterial growth and chemotaxonomy analysis—Bennett’s agar, ISP2, ISP4, Luria-Bertani (LB), marine agar (MA), nutrient agar (NA), potato dextrose agar (PDA), Reasoner’s 2A agar (R2A) and trypticase soy agar (TSA)—were provided by BD Difco (Becton, Dickinson and Co., Sparks, MD, USA). Other than those mentioned specifically, all chemicals were purchased as analytical grade from Sigma-Aldrich (Sigma-Aldrich, St. Louis, MO, USA).

### 2.2. Bacteria and Culture Condition

AN120528^T^ was isolated in 2012 from soil at Goesan-gun, Chungcheongbuk-do, Republic of Korea (36°44′0.6″ N, 127°51′30.1″ E) by spreading on R2A at 28 °C and with a five-day incubation. The isolate was maintained as glycerol (20%) stock solution at −75 °C.

### 2.3. Phylogenetic Analysis

The gDNA was isolated using a FastDNA SPIN Kit (MP Biomedicals Korea, Seoul, Korea). The 16S rRNA gene was amplified with a 27F-1492R primer set and sequenced using BigDye(R) Terminator v3.1 Cycle Sequencing Kits (Applied Biosystems, Foster City, CA, USA). The sequence similarities were compared using the GenBank database and EzBioCloud (https://www.ezbiocloud.net (accessed on 5 January 2022)) server. Phylogenetic distances were calculated by using the neighbour-joining [8,9], maximum-likelihood [10] and maximum-parsimony methods [11] in the MEGA 7.0 program, with bootstrap support values based on 1000 replications [12].

### 2.4. Genome Sequencing and Genomic Analysis

The preparation of gDNA for sequencing was performed by MGIEasy DNA Library Prep Kit (BGI, Shenzhen, China) on the de novo MGI platform. The resulting reads were quality trimmed to the Q_30_ confidence level. The read sequences (12× coverage) were assembled with the CLC Assembly Cell 5.1.1 (Qiagen Inc, Cambridge, MA, USA) using default parameters. The sequences were deposited in the National Center for Biotechnology Information (NCBI) GenBank under accession numbers JAKWBN000000000. The draft genomes were annotated by Rapid Annotation using Subsystem Technology 2.0 (RAST; https://rast.nmpdr.org (accessed on 10 May 2022)) [13]. The circular map was constructed using the PATRIC 3.5.43 online server for bacteria [14]. ANI values were calculated using OrthoANI software [15].

Based on the genome sequence, the gDNA G + C content and the estimated digital DNA–DNA hybridisation (dDDH) values were calculated using the Genome-to-Genome Distance Calculator (GGDC 2.1, https://ggdc.dsmz.de/ggdc.php (accessed on 10 June 2022)) [16]. The functional metabolite BGCs were analysed with the bacterial version of the antiSMASH v5.1.0 software (Technical University of Denmark, Kongens Lyngby, Denmark) [17]. The antibiotic resistance genes were analysed by performing a BLAST search against the Comprehensive Antibiotic Resistance Database (CARD; https://card.mcmaster.ca (accessed on 10 June 2022)). All the microbe protein (FAA) data were retrieved from the NCBI and the phylogenomic trees were constructed using the CVTree 3.0 (http://cvtree.online/v3/cvtree/ (accessed on 20 June 2022)).

### 2.5. Morphological, Phenotypic and Physiological Analysis

Gram-staining was performed using a Gram-stain kit according to the manufacturer’s instructions (bioMérieux, Lyon, France). Cell morphology was observed by scanning electron microscopy (SEM; JSM-6490LV, JEOL, Tokyo, Japan). For the carbon utilisation test, the basal medium consisted of the following: 2.64 g (NH_4_)_2_SO_4_, 5.65 g/L K_2_HPO_4_, 2.38 g/L KH_2_PO_4_, 1.0 g/L MgSO_4_·7H_2_O, 0.0079 g/L MnCl_2_·4H_2_O, 0.0064 g/L CuSO_4_·5H_2_O, 0.0015 g/L ZnSO_4_·7H_2_O, 0.0011 g/L FeSO_4_·7H_2_O and pH 7.2–7.4. The tested carbon sources included lactose, saccharose, mannitol, inositol, xylose, arabinose, glucose, fructose, rhamnose, galactose, mannose and starch. They were added to the basal medium at 0.5%, respectively. For the nitrogen source utilisation test, the basal medium consisted of the following: 1 g _D_-glucose, 5.65 g/L K_2_HPO_4_, 2.38 g/L KH_2_PO_4_, 1.0 g/L MgSO_4_·7H_2_O, 0.0079 g/L MnCl_2_·4H_2_O, 0.0064 g/L CuSO_4_·5H_2_O, 0.0015 g/L ZnSO_4_·7H_2_O, 0.0011 g/L FeSO_4_·7H_2_O and pH 7.2–7.4. Then, 0.5% of each nitrogen source was added to the basal medium. The tested nitrogen sources included arginine, _L_-glutamic acid, _L_-aspartic acid, _L_-cysteine, _L_-lysine, _L_-tyrosine, _D_-valine, _L_-isoleucine, _L_-serine, glycine, _L_-cysteine, _L_-alanine and _L_-threonine. After inoculation of the cells, observation continued for four weeks. The development of spores was examined as described previously using SEM in the aerial mycelium on the tested media after cells had grown on R2A for 30 days [18,19]. Anaerobic growth was examined on the R2A agar medium using a GasPak EZ Anaerobe Pouch System (Beckton Dickinson and Company, Sparks, MD, USA) at 28 °C. Cell motility was observed by using a phase-contrast microscope (Nikon SMZ-U, Tokyo, Japan), following the hanging-drop technique [20]. The tests included growth temperature range (4–55 °C), different concentration of NaCl (0–10%, *w*/*v*) and pH range (pH 4.0–11.0). The media pH was adjusted using 1 M Na_2_HPO_4_/NaH_2_PO_4_ or Na_2_CO_3_/NaHCO_3_ buffers. Catalase activity was examined with 3% (*v*/*v*) hydrogen peroxide solution and evaluated by observation of bubble formation. Oxidase activity was tested using oxidase reagent from an API 20E kit (bioMérieux, Lyon, France) under the manufacturer’s instructions. The antibiotic susceptibility was determined on R2A utilising paper discs containing the following antibiotics (µg per disc): amikacin (30), ampicillin/sulbactam (20), chloramphenicol (30), erythromycin (30) gentamicin (30), kanamycin (30), lincomycin (15), rifampicin (30), spectinomycin (25), streptomycin (25), teicoplanin (30), tetracycline (30) and vancomycin (30) [21,22]. Other metabolic properties were analysed using API 50CH and API ZYM kits (bioMérieux, Lyon, France) according to the manufacturer’s instructions. Coagulation and peptonization on skimmed milk were observed for 30 days after microbe inoculation. The test medium contained 200 g of skimmed milk and 0.2 g of CaCO_3_ and was autoclaved twice at 115 °C for 10 min [23]. Cells were inoculated into a medium containing gelatin (20%) and gelatin liquefaction was observed for 30 days. Starch degradation was tested on media containing soluble starch (1%) by halo zone detection after being staining with I_2_-KI solution (0.15% I_2_ in 1.5% KI).

### 2.6. Chemotaxonomy

The cells of strain AN120528^T^ were harvested from the culture broth and grown on TSA medium at 28 °C for three days. The fatty acids were extracted and methylated according to the instructions of the Microbial Identification System (MIDI) [24] and analysed with a gas chromatography (Model 6890; Hewlett Packard Co., Wilmington, DE, USA). Menaquinones were analysed by high-performance liquid chromatography (HPLC) according to Tamaoka et al., (1983) [25]. Cell wall amino acids and sugars were identified by following the method described in Staneck and Roberts (1974) [26]. Polar lipids were examined by two-dimensional thin-layer chromatography (TLC, silica, 20 × 20 cm, Merck, Darmstadt, Germany). For the first and second dimensional separation, a mixture of chloroform:methanol:DW (65:25:4, *v*/*v*) and another mixture of chloroform:acetic acid:methanol:DW (80:18:12:5, *v*/*v*) were used, respectively. Cell harvests were hydrolysed in 1.0 M sulfuric acid at 100 °C for 6 h and used for whole-cell sugar component analysis. The extracts were loaded onto cellulose TLC plates and developed by a mixture solution of n-butanol:DW:pyridine:toluene (10:6:6:1, *v*/*v*), twice [27].

### 2.7. Molecular Modelling and Docking

The molecular modelling and docking based on amino acid sequences were performed with reference to the previous study [28]. Briefly, amino acid sequences of putative biosynthesis-related enzymes were applied to the protein structure homology-modelling server (https://swissmodel.expasy.org/ (accessed on 11 July 2022)) [29]. Then, TfuA-Leader (PDB ID: 6jx3.1.B), hypothetical protein Atu2299 (2hly.1.A), asparagine synthetase (6gq3.1.A), TfuB1 lasso peptide synthetase B1 (6jx3.1.A) and uncharacterised ABC transporter ATP-binding protein TM_0288 (6quz.1.B) were used as templates for crystal structures. The three-dimensional structures were further analysed using PyMOL (DeLano Scientific, San Carlos, CA, USA, https://www.pymol.org (accessed on 11 July 2022)). Open Babel 2.4.0 (OpenEye Scientific Software, Inc., Santa Fe, NM, USA) was used to optimise the geometry and to minimise the energy of molecules [30]. Each three-dimensional structure was further modified and visualised using the molecular modelling program (https://www.cgl.ucsf.edu/chimera (accessed on 12 July 2022)). For docking analysis, the partial structures of the lasso peptides were sketched using ACD/ChemSketch 12.01 software (www.acdlabs.com (accessed on 12 July 2022), Toronto, ON, Canada), and then the sketched peptides were optimised using gradient optimisation after addition of explicit H atoms. Possible interactions of processing enzymes with the peptide were evaluated using the SwissDock server (SIB Swiss Institute of Bioinformatics, Lausanne, Switzerland) [31]. Intramolecular interactions were estimated by the Protein Interactions Calculator (PIC; (https://crick.mbu.iisc.ernet (accessed on 13 July 2022). in/∼PIC) [32].

## 3. Results

### 3.1. Phylogenetic Analysis

**Figure 1:**
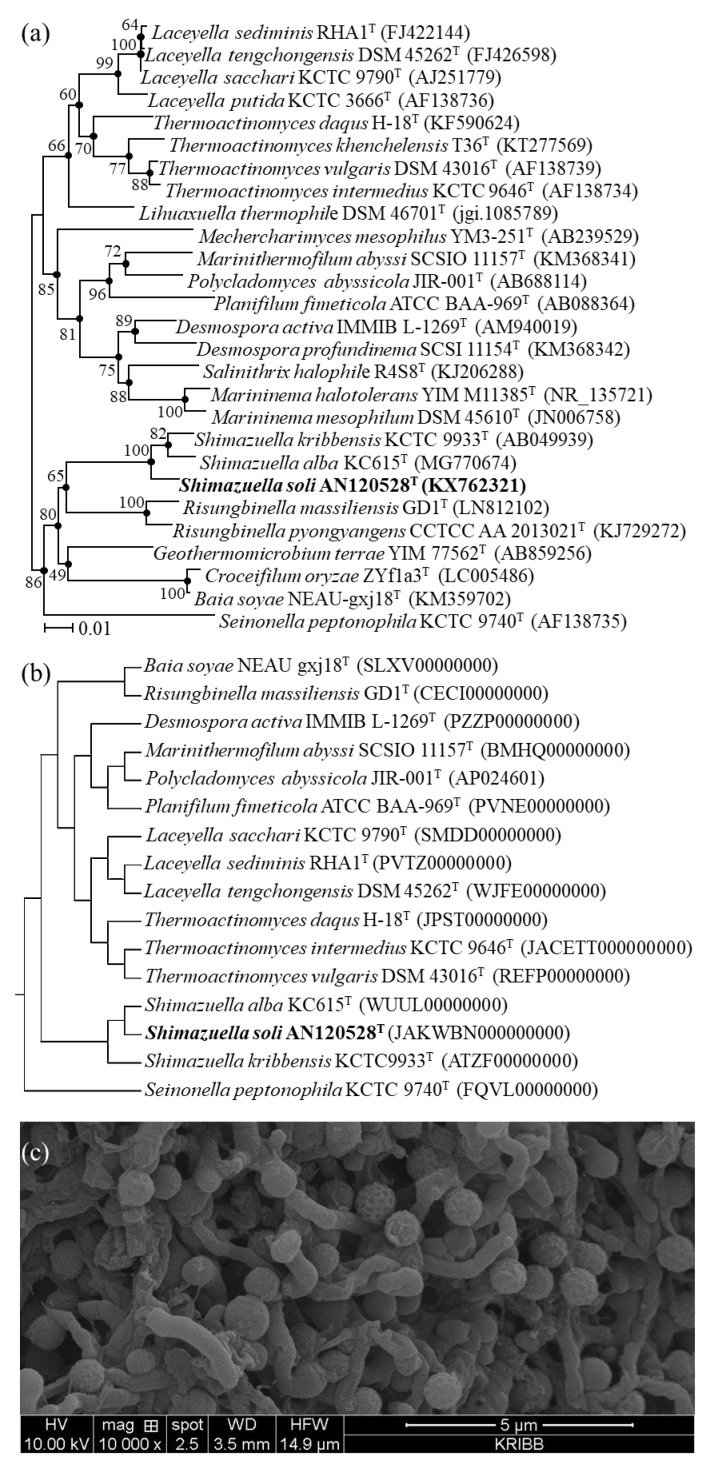
Phylogenetic tree, phylogenomic tree and SEM of the strain AN120528^T^. (**a**) Neighbour-joining phylogenetic tree showing the relationships of the strain AN120528^T^ with *S*. *alba* KC615^T^, *S*. KCTC 9933^T^ and other related species in the *Thermoactinomycetaceae* family. Bootstrap percentages (50%) based on 1000 re-samplings are given at the nodes. Dots indicate branches that were also recovered using the maximum-parsimony and maximum-likelihood algorithms. Bar, 0.01 substitutions per nucleotide position. (**b**) Phylogenomic tree of the genus *Shimazuella* and related genera. (**c**) SEM of the strain AN120528^T^ grown on an R2A agar plate for 5 days at 28 °C.

Phylogenetic and phylogenomic analysis based on the 16S rRNA gene (1470 bp; GenBank accession number KX762321) and genome sequence revealed that the strain AN120528^T^ was clearly a member of the genus *Shimazuella*, having the highest 16S rRNA gene sequence similarity to *S*. *alba* KC615^T^ (98.11%; accession no. MG770674) [4], followed by *S*. *kribbensis* KCTC 9933^T^ (97.05%; AB049939) [2] (Figure 1a,b). In addition, the neighbour-joining, maximum-parsimony and maximum-likelihood tree-making algorithms showed that the strain AN120528^T^ formed a monophyletic group with the two species of the genus *Shimazuella*. 

### 3.2. Morphological, Physiological and Biochemical Characteristics

The strain AN120528^T^ could grow well on Bennett’s agar ISP2, PDA and R2A. The strain showed a white-coloured colony and a 1.0 × 1.2 μm cell size on R2A agar medium (Figure 1c). Cells were aerobic and non-motile with a white colour. The strain AN120528^T^ was gram-positive, aerobic, spore-forming and non-motile. Colonies were circular, opaque and creamy white on the R2A medium. AN120528^T^ was able to grow in the range of 20–45 °C, with optimum growth at 28–40 °C, and at a pH of 6.0–7.0 with an optimum pH of 7.0, and it could tolerate up to 1.0% NaCl. Anaerobic growth was not observed. For cell growth, the strain utilised _D_-galactose and _D_-mannose as carbon sources and _L_-tyrosine and _L_-cysteine as nitrogen sources. It was positive for Tween 40 and 80 and negative for Tween 20. Cells were susceptible to all antibiotics tested. Table 1 shows several characteristics that distinguish the strain AN120528^T^ from the phylogenetically closely related strains.

### 3.3. Chemotaxonomic Characteristics

**Figure 2:**
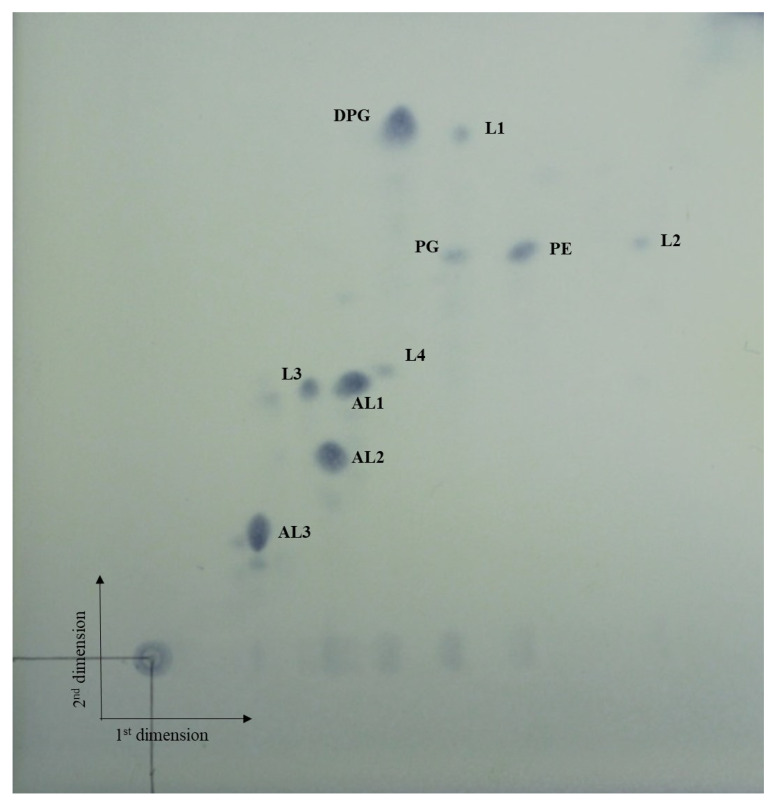
TLC of the polar lipids of strain AN120528^T^. Spots were stained with 5% ethanolic molybdophosphoric acid.

Table 2 lists the chemotaxonomic characteristics of the strain AN120528^T^ and its related strains. The major fatty acids (>30%) of AN120528^T^ were *anteiso*-C_15:0_ (32.3%) and *iso*-C_15:0_ (31.8%). Furthermore, the strain possessed MK-9 (H_4_) and MK-10 (H_4_), as the predominant menaquinones, and meso-diaminopimelic acid in the cell-wall peptidoglycan. The major polar lipids were DPG, PG, PE, AL and L (Figure 2). The major cell-wall sugars were ribose and glucose.

### 3.4. Genome Analysis

**Figure 3:**
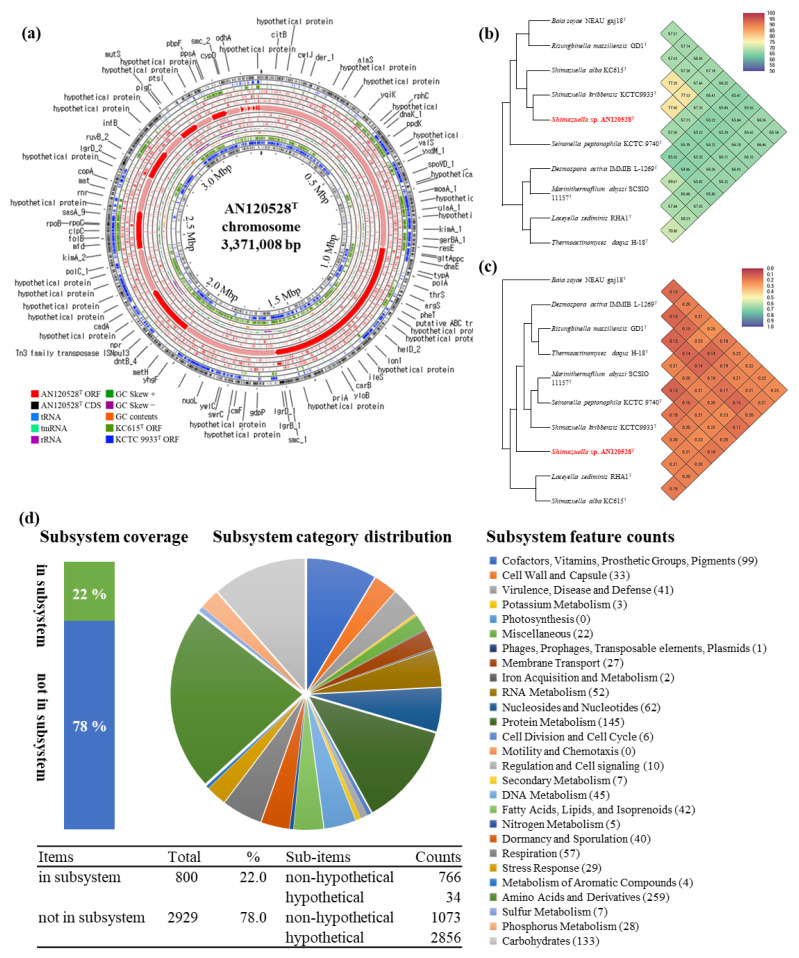
Graphic representation of genomic features of AN120528^T^. (**a**) Circular plot of the comparison genomes of AN120528^T^, KC615^T^ and KCTC 9933^T^. (**b**) OrthoANI analysis with other related strains. (**c**) GGDC analysis. (**d**) Genome annotation results of AN120528^T^ on the RAST webserver.

The assembled draft genome of AN120528^T^ was 3.37 Mbp, containing 25 contigs with an N50 length of 408,672 bp, 3408 coding sequences, 10 rRNA and 52 tRNA (Table 3). The gDNA G + C content was revealed to be 39.0%. The GenBank accession number for the genome sequences of the AN120528^T^ strain is JAKWBN000000000. Figure 3a shows the comparative genomic circular map. The OrthoANI values between AN120528^T^ and its related species *S*. *alba* KC615^T^ (accession no. WUUL00000000) and *S*. *kribbensis* KCTC 9933^T^ (ATZF01000001) were 77.53 and 77.60%, respectively (Figure 3b). Furthermore, the dDDH values of *S*. *alba* KC615^T^ and *S*. *kribbensis* KCTC 9933^T^ were 20.60 and 21.13%, respectively (Figure 3c). Based on the genome analysis on the RAST webserver, around 22% of detected genes—a total of 800 genes—were annotated in the subsystem (Figure 3d). The antiSMASH results on the functional metabolites showed BGCs for the one lasso peptide, three terpenes, one type I polyketide synthase (T1PKS), one type III polyketide synthase (T3PKS), three non-ribosomal peptide synthetases and one azol(in)e-containing peptide.

### 3.5. In Silico Analysis of the Novel Lasso Peptide Shimazuellin of AN120528^T^

#### 3.5.1. Genome Mining and Identification of Shimazuellin BGCs

**Figure 4:**
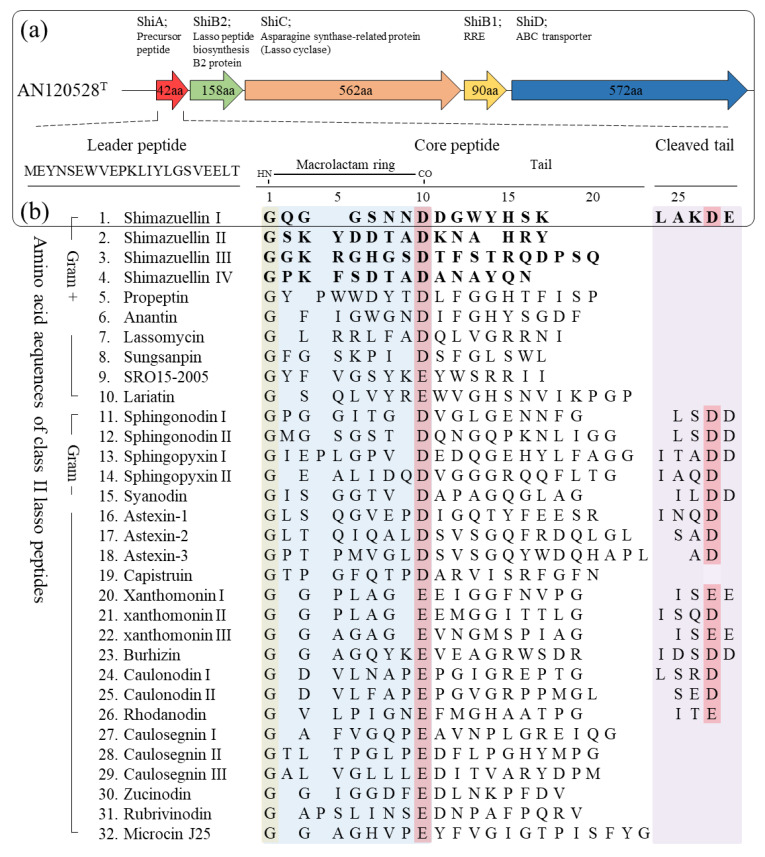
Gene organisation in the lasso peptide cluster of AN120528^T^ and other core peptide sequences of class II lasso peptides. (**a**) Shimazuellin biosynthetic gene locus. (**b**) The putative leader and core regions of Shimazuellin with other related lasso peptides.

Based on the precursor peptide sequence (protein ID; 00326) predicted from antiSMASH, shimazuellin was identified as a class II lasso peptide, and was called shimazuellin due to it being the first lasso peptide discovered in the genus Shimazuella. Adjacent proteins (00327, 00328, 00329 and 00330) of the precursor estimated to be essential enzymes in the biosynthetic pathway were selected as queries, and a BLAST homology search was performed (Table 4). The putative precursor peptide sequence was shown to have low levels of similarity with hypothetical protein PPOP_1752 (GAC42395.1) and hypothetical protein PPOP 1273 (GAC41916.1) of *Paenibacillus popilliae* ATCC 14706 at 47.06% and 45.45%, respectively. The putative lasso peptide-related proteins, which were annotated as uncharacterized proteins, had the highest amino acid sequence similarity to the lasso peptide biosynthesis protein (67.09%; accession no. WP_028776449.1), the asparagine synthase-related protein (65.54%; WP_028776448.1) and the hypothetical protein (60.70%; WP_028776447.1) of the strain KCTC 9933^T^. Although they had low sequence similarities with even closely related proteins, lasso-related enzymes were identified by the local presence of gene encoding proteins matching the pivotal motif and domain for the precursor peptide, the lasso protease, the lasso cyclase, the RiPP recognition element (RRE) and the ABC transporter. The shimazuellin BGC housed the five major genes involved in peptide precursor, biosynthesis, maturation and secretion. Although the sequences and functions of the peptides can vary markedly, the BGC for shimazuellin has a typical lasso peptide biosynthetic gene locus encoding a linear precursor peptide without disulfide bonds, three conserved proteins for peptide maturation and a transporter to export the matured peptide (Figure 4a). ShiA consists of 22 amino acids for the leader peptide, 16 amino acids for the core peptide region, and five amino acids for the truncated C-terminal tail, respectively [34]. The shiC encodes for amidotransferase, ATP pyrophosphatase and asparagine synthetase-like protein domain, which is responsible for formation of both isopeptide bonds and subsequent macrocyclisation [35]. ShiB1, containing a pyrroloquinoline quinone protein domain D (PqqD), also known as the RRE, binds to the leader region and transfers the precursor peptide to the protease ShiB2 for further processing [34,36]. The ShiD is an ABC transporter which secretes shimazuelin from the cytoplasm to the extracellular space, and the presence of the ShiD indicates that the biosynthesised lasso peptides could exhibit antimicrobial activity. The putative leader region, MEYNSEWVEPKLIYLGSVEELT, was shown to have a VXPXLXXXG conserved motif, which is commonly found in lasso leader peptides [37]. The leader and core regions are separated by the emblematic TG motif needed to remove the leader peptide during maturation [38]. Residues G and D were identified in the core peptide which can form macrolactam rings [39] (Figure 4b). Furthermore, a cleaved tail containing D residues was identified. This is not commonly found in gram-positive bacteria. In addition, in this study, class II lasso peptides that do not have a cleaved tail were found in *S*. *alba* KC615^T^ (shimazuellin II and IV) and *S*. *kribbensis* KCTC 9933^T^ (shimazuellin II and shimazuellin III) using comparative genome analysis.

#### 3.5.2. Scheme of the Putative Biosynthetic Mechanism of Shimazuellin in AN120528^T^

**Figure 5:**
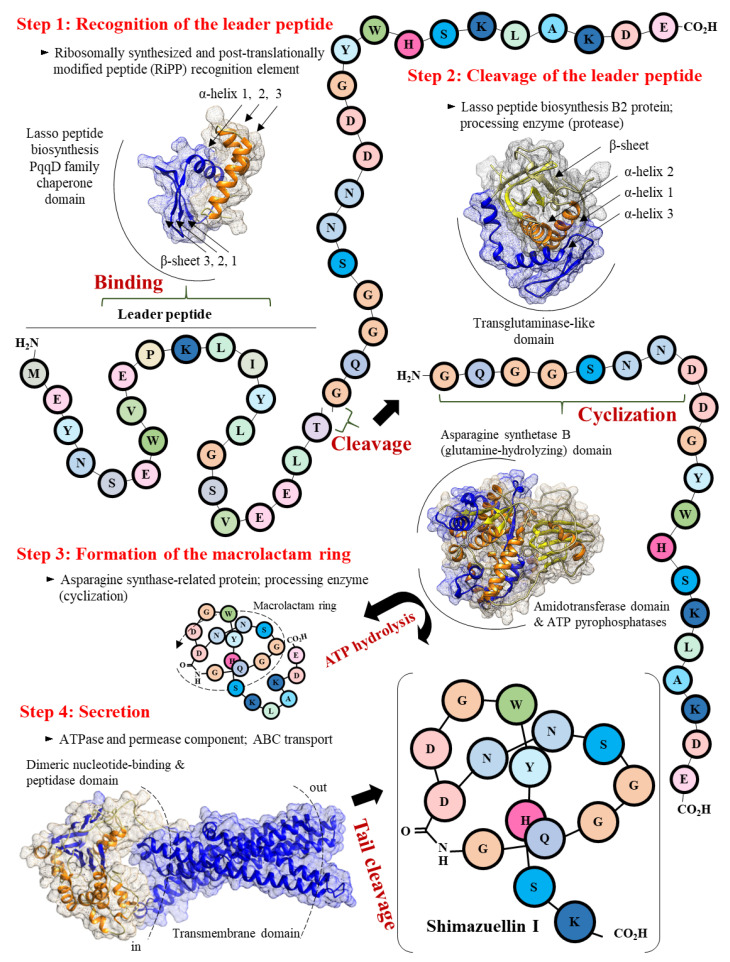
Proposed mechanism of shimazuellin biosynthesis and secretion, involving four steps.

Figure 5 shows the proposed mechanism of shimazuellin biosynthesis and secretion. Four steps seem to be necessary for the biosynthesis and secretion of shimazuellin based on the gene clusters encoding separate ShiA, ShiB2, ShiC, ShiB1 and ShiD. First, after a precursor peptide of shimazuellin is translated from mRNA, ShiB1 binds to the VXPXLXXXG region of the leader peptide in ShiA to recognise ShiA. Second, the ShiB2 protein removes the leader peptide via proteolysis of the TG region and thereby releases the core peptide of shimazuellin. Next, ShiC, the lasso cyclase, activates the Asp carboxylic acid in the form of an adenosine monophosphate ester before catalysing the macrolactam formation via condensation with the α-amino group. Finally, the ShiD-encoded ABC transporter performs cleavage in the tail (-LAKDE-) and exports the mature form of shimazuellin out of the cells.

### 3.6. Description of Shimazuella Soli Sp. Nov.

*Shimazuella soli* (so’li. L. neut. gen. n. soli of soil, referring to the source of the strain) cells are gram-positive, spore-forming, non-motile and white coloured. Growth occurs at 20–45 °C (optimum, 28–40), with a pH of 6.0–7.0 (optimum, 7.0) and in the presence of 0–1% (*w*/*v*) (optimum, 0.5) NaCl. The strain utilises _D-_galactose and _D-_mannose as carbon sources and _L-_tyrosine and _L-_cysteine as nitrogen sources for growth. It is susceptible to amikacin, ampicillin/sulbactam, chloramphenicol, erythromycin, gentamicin, kanamycin, lincomycin, rifampicin, spectinomycin, streptomycin, teicoplanin, tetracycline and vancomycin. It can degrade Tween 40 and 80, but not Tween 20, starch, cellulose or gelatine. It can solidify and peptonise milk. It is positive for β-glucosidase. Its major fatty acids are *iso*-C_14:0_, *iso*-C_15:0_, *anteiso*-C_15:0_ and *iso*-C_16:0_. The respiratory quinones from the cell wall are MK-9 (H_4_) and MK-10 (H_4_). Its polar lipids are diphosphatidylglycerol, phosphatidylglycerol, phosphatidylethanolamine, unidentified aminolipid and unidentified lipids. The G+C content of the gDNA is 39.0%. The type strain, AN120528^T^ (=KCTC 39810^T^ = DSM 103571^T^), was isolated from soil in South Korea.

## 4. Discussion

The genus *Shimazuella*, represented by *S*. *kribbensis* KCTC 9933^T^, was first proposed in 2007 [2]. However, the *Shimazuella* spp. have consisted of only two recognized species so far. Based on our observation, along with previously published results, *Shimazuella* spp. prefer extremely limited or unique carbon sources for metabolism and take about 7 days or more to reach the stable late log phase or early stationary phase [2,4]. *Shimazuella* is considered to have evolved a metabolism that prefers other sugars and does not have a glucose metabolism, which is used as a basic carbon source for most organisms to survive in a community consisting of various microbes in soil. Because of these physiological characteristics, it could be difficult to isolate by antagonistic actions in the microbial community during the screening process of a single strain from the environment. Furthermore, the limitations of *Shimazuella* isolation were supported by an antibiotic susceptibility test and genome analysis in this study. These analyses found that *Shimazuella* spp. do not contain any antibiotic resistance genes except for one putative glycopeptide resistance gene cluster common to all strains and two antibiotic efflux-related genes in the strain KC615^T^. In this study, we successfully isolated AN120528^T^ from soil using extreme serial dilution to obtain rare bacteria and investigate their functionality. Comparative genome analysis showed that the ANI and dDDH values between the strain AN120528^T^ and its related species were lower than the cut-off of 95–96% and were 70% for the delineation of a novel species, respectively [40,41]. Moreover, the results of the carbon utilisation and fatty acid composition allowed for the systematic differentiation of AN120528^T^ from related species. Therefore, the strain AN120528^T^ represents a novel species of the genus *Shimazuella*, for which we propose the name *Shimazuella soli* sp. nov. In addition, the description of the genus *Shimazuella* is as given previously [2,4], with the following modifications: its diagnostic polar lipids are DPG, PE and PG; its major fatty acids are *anteiso*-C_15:0_, *iso*-C_14:0_, *iso*-C_15:0_, *iso*-C_16:0_ and C_16:0_; and the G+C content is around 38.4–39.0%.

Shimazuellin I, a new lasso peptide belonging to class II in *S*. *soli* AN120528^T^, was discovered, and lasso peptides with different sequences were also found in KC615^T^ (shimazuellin II and IV) and in KCTC 9933^T^ (shimazuellin II and shimazuellin III). Although its sequence homology with known lasso peptide biosynthetic enzymes was significantly low, sequence-based protein 3D modelling, comparative structure analysis, conserved domain and in silico molecular docking analysis demonstrated that the ShiA, ShiB1, ShiB2, ShiC and ShiD enzymes could be involved in lasso peptide biosynthesis. Shimazuellin I of *S*. *soli* AN120528^T^ was identified for the first time in this study, as it has a C-terminal cleavage tail unlike lasso peptides generally reported in gram-positive bacteria. In addition to shimazuellin I–IV, the following putative BGCs for various antimicrobial peptides were identified: enniatin, micrococcin P1, non-ribosomal tripeptide (D-Phe-D-Ala-Trp) and carnocyclin A in *S*. *soli* AN120528^T^; xenematide, lanthipeptide, micrococcin P1, sevadicin and xenotetrapeptide in KC615^T^; and micrococcin P1 and massetolide A in KCTC 9933^T^. It is inferred that *Shimazuella* possesses various antimicrobial peptides for the stable uptake of nutrients after it germinates in the presence of sufficiently preferred sugars, even though it maintains a spore state under unfavourable conditions. This is similar to how *Shimazuella* has evolved a unique and limited carbohydrate metabolism to survive.

The trend in peptide science has changed from simply finding and applying natural peptides derived from organisms to the rational design of peptides with desirable physiological functions [42]. Major innovations in genomics, bioinformatics, and sequencing technology have enabled the rational design of excellent peptides with desirable biochemical activities. As a result, approximately 20 new therapeutic peptides have been released in the last 10 years, and dozens of peptides are in clinical development [43]. Therefore, the continuous discovery of new peptides that can inspire us makes peptides applicable to a wide range of diseases. At present, the existence of many lasso peptide gene clusters has been identified from a variety of bacteria with the development of bioinformatics and next-generation sequencing technology, resulting in revealed amino acid sequence diversity of precursor peptides [44]. Based on their biological functions, the peptides may be useful tools for the treatment of metabolic syndrome, autoimmune disease, microbial infections and cancer [7,45,46]. Furthermore, the macrocyclic forms of lasso peptides are an appropriate backbone for epitope grafting due to their proteolytic stability and thermostability [47]. These redesigned peptides can be applied as molecular probes and drug carriers for therapeutics [48,49,50]. Therefore, in addition to determining the extent of their physiological function, the major motivations of genomic efforts are to mine new examples of lasso peptides and discover novel classes, such as shimazuellin found in *S*. *soli* AN120528^T^ in our study. Considering the wide selection of active candidates, we suggest that the mining of shimazuellin can both contribute to an expansion in the scope of peptide therapeutics and be used in basic research that advances our peptide design capabilities. Taken together, we believe that the *S*. *soli* AN120528^T^ and shimazuellin we report here could be utilised as useful information to boost peptide research in the postgenomic era.

## Figures and Tables

**Table 1:** Physiological and biochemical properties of the strain AN120528^T^ and related strains. All strains were gram-positive, aerobic and non-motile, and anaerobic growth was not observed. All strains also have MK-9 (H_4_) and MK-10 (H_4_) as respiratory quinones, meso-diaminopimelic acid as the diamino acid peptidoglycan and ribose and glucose as the major cell-wall sugars.

Characteristics	AN120528^T^	KC615^T^	KCTC 9933^T^
Spores (μm)	1.1–1.2	0.6–0.9	1.0–1.4
Growth conditions			
Temperature range (°C)	20–45	28–37	20–50
pH range	6.0–7.0	6.0–8.0	6.0–9.0
NaCl tolerance (%)	0–1	0–1	0–2
Degradation of			
Starch	–	–	+
Gelatin	–	–	–
Tween 40	+	+	–
Tween 80	+	+	–
Carbon utilisation			
_D_-Galactose	+	–	–
_D_-Mannose	+	–	–
_D_-Raffinose	–	+	–
Adonitol	–	+	–
Nitrogen utilisation			
_L_-Alanine	–	+	–
_L_-Arginine	–	+	–
_L_-Asparagine	–	–	+
_L_-Cysteine	+	+	–
_L_-Methionine	–	–	+
_L_-Tyrosine	+	–	+
_L_-Valine	–	+	–
Enzymatic assay			
Arbutin hydrolysis	–	+	–
β-glucosidase	+	–	–
Major polar lipids *	DPG, PE, PG, 3AL, 4L	DPG, PE, OH-PE, AL, GL, L	DPG, PE, PG, PME, APL, 4AL, 2L

DPG, diphosphatidylglycerol; PG, phosphatidylglycerol; PME, phosphatidyl-N-methylethanolamine; PE, phosphatidylethanolamine; AL, aminolipid; OH-PE, hydroxy-phosphatidylethanolamine; APL, unknown aminophospholipid; GL, glycolipid; L, unknown lipid. * The polar lipids of KC615^T^ and KCTC 9933^T^ were obtained from Saygin et al. (2020) and Kim et al. (2015) [4,33].

**Table 2:** Fatty acid compositions of strain AN120528^T^ and another related strains.

Fatty acid (%)	AN120528^T^	KC615^T^	KCTC 9933^T^
Saturated			
C_13:0_	tr	-	-
C_14:0_	1.1	1.4	1.4
C_16:0_	4.9	1.7	6.3
C_17:0_	1.4	-	tr
C_18:0_	1.1	10.1	-
C_19:0_	1.1	1.1	-
C_20:0_	-	15.8	-
Branched			
*iso* C_13:0_	tr	-	tr
*iso* C_14:0_	9.5	5.2	5.5
*iso* C_15:0_	31.8	6.2	13.2
*iso* C_16:0_	9.3	2.4	4.4
*iso* C_17:0_	1.5	2.7	1.1
*iso* C_18:0_	-	2.5	-
*iso* C_19:0_	-	4.9	-
*iso* C_17:1 ω10c_	tr		-
*anteiso*-C_13:0_	tr	-	-
*anteiso*-C_15:0_	32.3	37.91	59.1
*anteiso*-C_17:0_	1.5	5.1	3.2
*anteiso*-C_19:0_	-	3.3	-
C_16:1 *ω*11*c*_	1.8	-	3.4
C_16:1 *ω*7*c* alcohol_	1.9	-	5.5
C_18:1 *ω*9*c*_	Tr	-	-
Summed feature 4	-	-	tr

-, not detected. Summed feature 4 contains iso-IC_17:1_/anteiso-B or anteiso-C_17:1_ B/iso-I. tr, trace amount <1%. The fatty acids of KC615^T^ and KCTC 9933^T^ were obtained from Saygin et al. (2020) and Kim et al. (2015) [2,33].

**Table 3:** General genomic and functional features of AN120528^T^ and related members of the genus *Shimazuella*.

Features	AN120528^T^	KC615^T^	KCTC 9933^T^
Genome size (bp)	3,371,008	3,989,583	4,185,101
Contigs	25	44	42
N50 (bp)	408,672	278,159	244,454
L50	contig 3	contig 6	contig 6
Total genes	3548	4054	4273
Pseudogene	74	203	109
CDSs	3408	3796	4087
rRNA	10	3	20
tRNA	52	48	53
G+C content (%)	39.0	38.5	38.4
Antibiotic resistance gene clusters	1 (glycopeptide resistance)	3 (one glycopeptide resistance and two antibiotic efflux)	1 (glycopeptide resistance)
CRISPR system (Number of spacers)	Type III-B (13)	Type I-C (30)	Type I-C (17)
Number of BGCs	10	11	16

**Table 4:** Proposed functions of the open reading frames in the putative lasso peptide biosynthetic-related proteins of AN120528^T^.

In the Genome of AN120528^T^			NCBI Blast			Putative
Protein ID	Locus (Contig 1)	Annotation	Description (Accession No.)	Scientific Name	Identity (%)	Protein (Functions)
00326	295741_ 295869	hypothetical protein	hypothetical protein PPOP_1752 (GAC42395.1)	*Paenibacillus popilliae* ATCC 14706	47.06	ShiA (precursor peptide)
00327	295926_ 296402	hypothetical protein	lasso peptide biosynthesis protein (WP_028776449.1)	*S*. *kribbensis* KCTC 9933^T^	67.09	ShiB2 (B2 element; protease)
00328	296415_ 298103	hypothetical protein	asparagine synthase-related protein (WP_028776448.1)	*S*. *kribbensis* KCTC 9933^T^	65.54	ShiC (cyclase)
00329	298066_ 298338	hypothetical protein	hypothetical protein (WP_028776447.1)	*S*. *kribbensis* KCTC 9933^T^	60.70	ShiB1 (B1 element; RRE)
00330	298360_ 300078	putative ATP-binding protein	ABC transporter ATP-binding protein/ permease (WP_028776446.1)	*S*. *kribbensis* KCTC 9933^T^	74.17	ShiD (ABC transporter)

## Data Availability

The raw data supporting the conclusions of this article will be made available by the authors, without undue reservation.
